# First Pediatric Outpatient Parenteral Antibiotic Therapy Clinic in Indonesia

**DOI:** 10.3389/fped.2020.00156

**Published:** 2020-04-15

**Authors:** Aryono Hendarto, Nina Dwi Putri, Dita Rizkya Yunita, Mariam Efendi, Ari Prayitno, Mulya Rahma Karyanti, Hindra Irawan Satari, Sri Rezeki S. Hadinegoro, Monica Chan

**Affiliations:** ^1^Department of Child Health, Academic Medical Center, Faculty of Medicine, Cipto Mangunkusumo Hospital, Universitas Indonesia, Jakarta, Indonesia; ^2^Department of Paediatric Nursing, Cipto Mangunkusumo Hospital, Jakarta, Indonesia; ^3^Department of Infectious Diseases, Tan Tock Seng Hospital, Singapore, Singapore

**Keywords:** ceftriaxone, Indonesia, urinary tract infections, outpatient, antibiotic

## Abstract

**Background:** Infection remains a major pediatric health problem in Indonesia and usually leads to longer hospitalization due to the need for extended intravenous antibiotic administration. In developed countries, pediatric outpatient parenteral antibiotic therapy (P-OPAT) is well-established and proven to be safe and effective at reducing the length of hospital stay; however, data on low- and middle-income countries such as Indonesia remain limited. This P-OPAT service is new and the first service in Indonesia.

**Methods:** The medical records of patients attending Indonesia's first P-OPAT clinic between April 2015 and March 2017 were retrospectively investigated.

**Results:** During the 24-month period, 32 patients received treatment at the P-OPAT clinic, saving a total of 258 bed days. The majority of patients (*n* = 16; 50%) were diagnosed with urinary tract infection, followed by cellulitis (*n* = 4; 12.5%) and osteomyelitis (*n* = 4; 12.5%). Ceftriaxone was the most commonly used antibiotic (*n* = 16; 50%). All patients used a peripheral intravenous catheter and were sent home with this device. Twelve patients (37.5%) needed to change IV access more than once. None of the patients used elastomeric infusor device. The median duration of OPAT was 5 days (range 1–27 days). All patients were successfully treated with no recurrence after 30 days. One patient (3.1%) experienced drug-related complication and another one (3.1%) was readmitted due to an underlying medical condition. All the patients complied with P-OPAT schedules.

**Conclusions:** P-OPAT service offers a safe and effective option for the delivery of outpatient intravenous antibiotics in selected patients even in resource-poor settings.

## Introduction

In low- and middle-income countries (LMICs) such as Indonesia, infectious diseases remain the major cause of morbidity in pediatric patients, which commonly leads to hospitalization (1). Severe infections may require prolonged hospital stays due to the need for an extended period of IV antibiotic administration (2). Prolonged hospital stays can harm patients by increasing the risk of nosocomial infections, increasing the family's financial burden, and reducing the psychological well-being, especially in children (3, 4).

One of the measures aimed at decreasing the length of hospital stays, or even avoiding admission altogether, is to facilitate the early discharge by means of outpatient parenteral antibiotic therapy (OPAT) service (5). OPAT involves administration of IV antibiotics in outpatient clinics, home-based settings, or community healthcare facilities on patients who need IV antibiotics and lack a suitable oral antibiotic alternative (6). OPAT has been a well-established treatment option for many adult healthcare facilities in resource-rich countries (7). Although the first OPAT was started in pediatric patients (8). the treatment progress in the pediatric population has not been as fast as in adults (9) OPAT has been proven to be effective, efficient (10), and safe, with low complication rates in both adult and pediatric populations (11, 12). On the other hand, in LMICs, OPAT is not yet well-established.

Since 2014, Indonesia had started the National Health Insurance (NHI) policy, progressing to be a universal healthcare service covering all of the citizens of Indonesia. Since this is the first OPAT service in Indonesia, there was still no administrative regulation about this service. There are several challenges in establishing OPAT service in Indonesia. As an alternative to reduce hospital stay, OPAT service is not yet covered in the NHI service. There is also no elastomeric infusors available yet so the antibiotics must be administered in the hospital every day. Our hospital Cipto Mangunkusumo hospital (CMH) also received referred patients from outside of Jakarta. Many of the patients did not have a place to live near the hospital. This resulted in increased travel time or added resources to find a temporary place during the proposed OPAT course.

This study aimed to describe the experience of, and challenges encountered in, developing the first pediatric OPAT (P-OPAT) clinic in Indonesia by examining its outcomes and safety.

## Methods

### Setting

**Figure 1:**
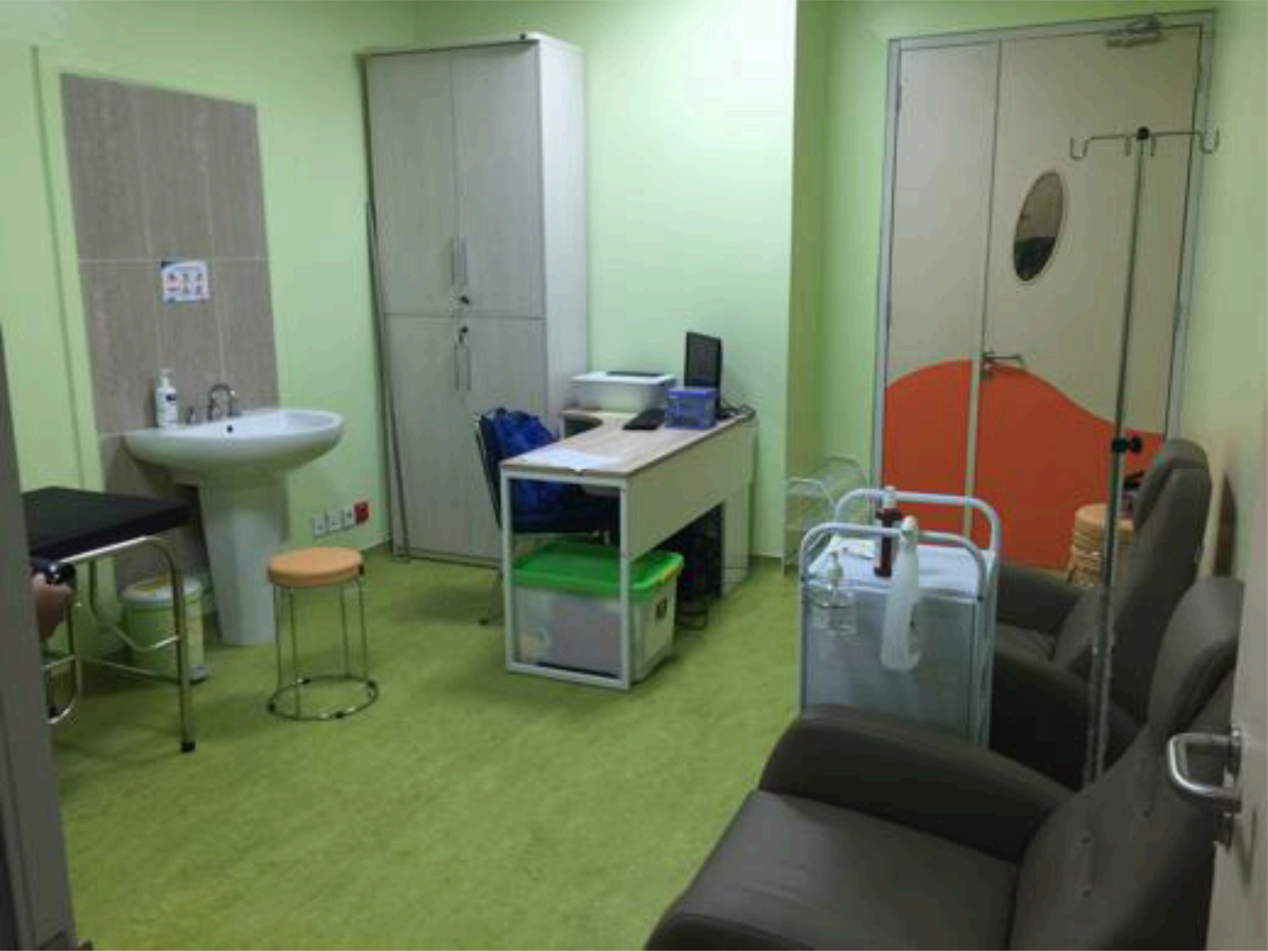
P-OPAT clinic in CMH.

This was a retrospective study performed in CMH, a tertiary hospital in Jakarta and also a national referral hospital in Indonesia. This hospital is a general hospital with 1,000 beds. There is a need for bed saving days since there is a constant waiting list for pediatric patients in CMH. The P-OPAT clinic in CMH was established in early 2015. P-OPAT clinic is located at Pediatric Unit of CMH. Our clinic has one bed and two treatment chairs (Figure 1). P-OPAT services are available every day from 8 a.m. to 4 p.m. During weekdays, P-OPAT service runs in P-OPAT clinic. While, during the weekend, P-OPAT services were delivered in the Emergency Department Treatment Center.

### Procedure

All patients referred to the P-OPAT clinic were from the inpatient ward and emergency department. They were assessed by at least one physician and one nurse. Patients had a clear diagnosis of infection, were clinically stable, and without suitable alternative oral antibiotic. The patient and family were adequately educated about routine care at home, P-OPAT schedule, complications, and emergency access. They were cooperative and confident to be discharged with further follow-up in P-OPAT clinic. Patients and/or their guardians provided informed consent for treatment in P-OPAT clinic. Patients who did not fulfil these criteria were excluded.

**Figure 2:**
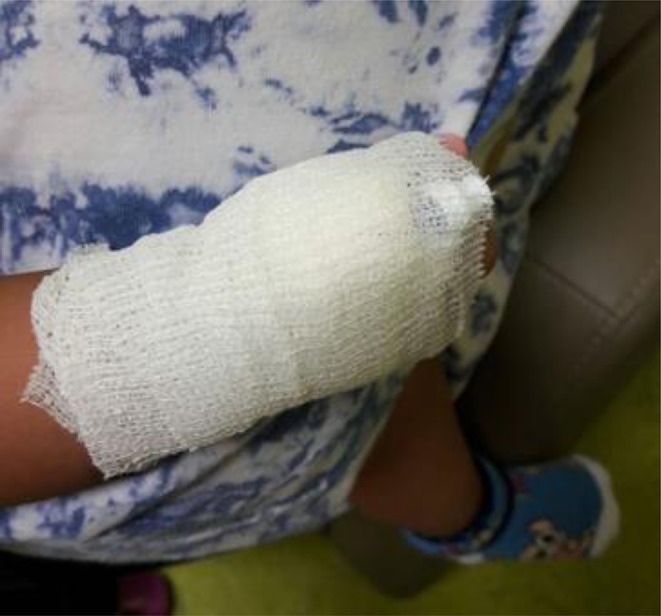
Safety of IV access using special bandage.

To ensure patient safety, all of the first doses of antibiotics were administered at the hospital. A 24-h emergency contact number was provided to assist patients during the P-OPAT service. Peripheral IV access was used in all patients with a neutral valve connector and covered by a bandage to ensure it was safe to bring home (Figure 2). Kits were also provided for every patient, consisting of small dressings, alcohol swabs, and sterile cottons. The P-OPAT guidelines were adopted and adjusted from Singapore and the United Kingdom for better implementation in our setting (9).

### Data Collection

This study was conducted through reviewing the medical records of patients who had received treatment in the P-OPAT clinic at CMH between April 2015 and March 2017. Demographic data, clinical characteristics, microbiological results, therapy received, and patient outcomes were obtained at treatment completion and 30 days after. The clinical outcomes consist of treatment rate, bed days saved, frequency of IV access replacement, and recurrence of infection for 30 days after treatment completion. Complications consist of a deterioration of infection, drug- and IV-access-related complications, and unscheduled medical visits. Bed days saved was defined as the total number of treatment days in the P-OPAT clinic. This study was approved by the Faculty of Medicine, University of Indonesia's Research, and Ethics Committee.

## Results

A total of 32 patients received P-OPAT service at CMH (Table 1). The majority (*n* = 31; 93.8%) of patients used national health insurance. Half of the patients lived outside Jakarta City, with a distance of more than 20 km from CMH. During the 2-year period, this service saved 258 bed days. The most common diagnosis was complicated UTI (*n* = 16; 60%), followed by cellulitis (*n* = 4; 12.5%) and osteomyelitis (4; 12.5%). During the second year period, we also start to take some difficult cases such as brain abscess (*n* = 1; 3.1%) and native valve endocarditis (*n* = 1; 3.1%). More than half of the diagnosis were unproven by culture (*n* = 19; 59.4%). Half of the patients were being treated with ceftriaxone (*n* = 16; 50%). All patients used peripheral IV access and were discharged home with the peripheral IV access (Table 2).

**Table 1:** Baseline demographics of P-OPAT patients.

**Characteristics**	**OPAT (*n* = 32)**	
	**Total**	**%**
**Sex**		
Male	17	53.1
Female	15	46.9
**Method of payment**		
Personal	2	6.3
Indonesian national health insurance	31	93.8
**Home address**		
Jakarta (location of P-OPAT clinic)	16	50.0
Bekasi (23 km from Jakarta)	6	18.8
Tangerang (25 km from Jakarta)	2	6.2
Other	8	25.0
**Referral**		
Outpatient clinic	5	15.6
Inpatient ward	18	56.3
Emergency room	9	28.1
**Age by group (years)**		
<1	4	12.5
1–5	11	34.4
6–10	7	21.9
>10	10	31.2
Age [median (range)] (months)	80 (4–192)	

**Table 2:** Clinical characteristics of P-OPAT patients.

**Characteristics**	**OPAT (*****n*** **=** **32)**	
	**Total**	**%**
**Diagnosis**		
Urinary tract infection	16	50.0
Cellulitis	4	12.5
Osteomyelitis	4	12.5
Native valve endocarditis	1	3.1
Abscess (mandibular and brain)	2	6.3
Fungal infection	3	9.4
Pneumonia	1	3.1
Other infection	1	3.1
**Microorganism**		
No growth	19	59.4
*Escherichia coli*	6	18.8
*Enterococcus faecalis*	2	6.3
*Klebsiella pneumonia*	1	3.1
*K*. *oxytoca*	1	3.1
*Staphylococcus aureus*	1	3.1
*Candida albicans*	1	3.1
*C*. *glabrata*	1	3.1
**Treatment**		
Ceftriaxone	16	50.0
Amikacin	8	25.0
Gentamicin	4	12.5
Micafungin	3	9.4
Ganciclovir	1	3.1
Duration of OPAT [median (range)] (days)	5 (1–27)	
Bed days saved	258	
**Intravenous access type**		
Central	0	0.0
Peripheral	32	100.0

All patients (*n* = 32; 100%) were clinically cured at the end of treatment (Table 3). No infections recurred within 30 days of discharge from P-OPAT. Twelve patients (37.5%) needed to change IV access more than once. One patient (3.1%) experienced drug-allergy-related complications during the first dose administered before hospital discharge to the P-OPAT clinic, and another (*n* = 1; 3.1%) was readmitted due to an underlying medical condition. The compliance rate in this study was 100%.

**Table 3:** Clinical outcomes and complications of P-OPAT service.

**Variables**	**Frequency (*****n*** **=** **32)**	
	**Total**	**%**
**Patient status at completion of OPAT**		
Clinically cured	32	100
Not cured	0	0
OPAT monitoring after 30 days		
Recurrence of infection	0	0
Continued follow-up for underlying medical condition	32	100
Death	0	0
**Frequency of peripheral IV access replacement**		
Never	6	18.8
1 time	14	43.8
2 times	5	15.6
3 times	3	9.4
4 times	1	3.1
≥5 times	3	9.4
**Complications**		
No complications	30	93.8
Deterioration of infection	0	0
Admission for the underlying medical condition	1	3.1
Drug-related side effects	1	3.1
IV access-related complications	0	0
Compliance	32	100

## Discussion

During the 2-year P-OPAT service in CMH, 258 bed days were saved in the treatment of 32 patients. Admissions were avoided in nine of 32 patients (28.1%) who attended the emergency room. The youngest patient treated at our clinic was 4 months old and completed P-OPAT without any complications. This finding is supported by a report from Utah, which found P-OPAT to be safe even for infants aged under 3 months (13).

Most patients at our P-OPAT clinic are diagnosed with complicated UTIs which were not responded with an oral antibiotic or no option of oral antibiotic. Diagnoses reported in other centers widely varied, but comprised mainly of bone, joint and soft tissue infections, UTIs, bloodstream infections, infections due to cystic fibrosis, and intra-abdominal infections (5, 11, 14). Two of our patients had endocarditis and brain abscess, diagnoses that usually require stricter criteria (9) for acceptance to P-OPAT. These patients had minimal residual abscess or vegetation, stable clinical condition, and residence in close proximity to the P-OPAT clinic. Studies in adults have shown no differences between the readmission and mortality rates in patients with endocarditis at the OPAT compared to inpatient treatment (15).

The complication rate of P-OPAT in our clinic was low (6.2%). One patient had an allergic reaction (urticaria) during the administration of the first dose of antibiotic, requiring changes in the type of antibiotics administered. The other patient required scheduled hospitalization for peritoneal dialysis device insertion due to underlying medical illness (chronic kidney disease).

All of our patients were discharged home with peripheral IV access. Ambulatory peripheral inserted central catheters (PICCs) are not yet available in Indonesia. Most centers use PICC and peripheral IV access in P-OPAT, but none have reported whether peripheral IV access was retained upon home discharge as used in our center (9, 11). During the 2-year period, more than 80% of patients experienced IV replacement of no more than twice, whereas the remainder required additional replacements due to occlusion. Any other complications, such as accidental removal, dislodgment, phlebitis, or infection during the treatment period, were not observed. Ceftriaxone was the most commonly used antibiotic, as reported by many other P-OPAT centers (5, 11, 13). However, a limitation was that most of the diagnoses were unproven by culture, making the treatment primarily empirical in nature.

In developing the first P-OPAT in Indonesia, several challenges were faced including the adaptation of the national insurance reimbursement financial model to support the P-OPAT; obtaining approval from multiple levels of hospital, regional, and national authorities; and promoting awareness and confidence in P-OPAT services among hospital physicians, patients, and patient's family. The extent of P-OPAT services in Indonesia was limited because of the lack of elastomeric infusors to deliver antibiotics that would otherwise require multiple daily dosing which is a standard practice in other OPAT centers (5, 9, 12). In Indonesia, the elastomeric infusor is still undergoing the national registration process; thus, this limits eligible patients to those requiring once daily single-dose antibiotics. The selection of antibiotics based on dosing convenience and availability should also be balanced against the role of OPAT in supporting the antibiotic stewardship program (16). Although our patients' parents prefer P-OPAT over hospitalization, approximately half of them came from outside the city of Jakarta, creating time inefficiencies that necessitate the consideration of alternatives, such as the introduction of OPAT services in community or district hospitals.

The strength of our study is this P-OPAT clinic is the first P-OPAT in Indonesia which is one of the LMICs. Besides that, we can show that with limited facilities, we can show that P-OPAT clinic still can run and help to save hospital bed days. In our study, we also showed that using peripheral IV catheter at home is safe for children. But in this study, we also have some limitations which is due to small number of patients and lack of P-OPAT service cost data.

In conclusion, our findings show that P-OPAT is safe and effective for selected pediatric patients and provides an opportunity to introduce additional P-OPAT services, as well as expanding the treatment to adult services in LMICs, such as Indonesia. The use of peripheral IV access can be an option for P-OPAT services in LMICs; however, more robust evidence is needed.

## Data Availability Statement

The raw data supporting the conclusions of this article will be made available by the authors, without undue reservation, to any qualified researcher.

## Ethics Statement

The studies involving human participants were reviewed and approved by Faculty of Medicine, University of Indonesia Research and Ethics Committee. Written informed consent to participate in this study was provided by the participants' legal guardian/next of kin. Written informed consent was obtained from the individual(s), and minor(s)' legal guardian/next of kin, for the publication of any potentially identifiable images or data included in this article.

## Author Contributions

AH, NP, and SH designed the study. AH, NP, DY, ME, AP, HS, and MK collected and analyzed data. AH, NP, SH, MK, and MC wrote the manuscript. HS, AP, MC, and SH provided technical support and conceptual advice. AH and NP obtained the research grant. All authors have read and approved the final manuscript.

### Conflict of Interest

The authors declare that the research was conducted in the absence of any commercial or financial relationships that could be construed as a potential conflict of interest.
